# The Involvement of Natural Polyphenols in the Chemoprevention of Cervical Cancer

**DOI:** 10.3390/ijms22168812

**Published:** 2021-08-16

**Authors:** Georgiana Drețcanu, Cristian I. Iuhas, Zorița Diaconeasa

**Affiliations:** 1 Faculty of Food Science and Technology, University of Agricultural Science and Veterinary Medicine Cluj-Napoca, Calea Mănăştur 3–5, 400372 Cluj-Napoca, Romania; georgiana.dretcanu@stud.ubbcluj.ro; 2 Faculty of Medicine, Iuliu Hatieganu University of Medicine and Pharmacy, 400372 Cluj-Napoca, Romania

**Keywords:** cervical cancer, HPV, polyphenols, phytochemicals, toxicity, apoptosis

## Abstract

From all types of cancer, cervical cancer manages to be in top four most frequent types, with a 6.5% rate of occurrence. The infectious vector that induces the disease, the high-risk Human papillomavirus (HPV), which is a sexually transmitted virus, is capable of transforming the host cell by modulating some of the principal signaling pathways responsible for cell cycle arrest, proliferation, and survival. Fortunately, like other cancer types, cervical cancer can be treated by chirurgical interventions or chemoradiotherapy, but these methods are not exactly the lucky clover of modern medicine because of the adverse effects they have. That is the reason why in the last years the emphasis has been on alternative medicine, more specifically on phytochemicals, as a substantial number of studies showed that diet contributes to cancer prevention and treatment. All these studies are trying to find new chemopreventive agents with less toxicity but high effectiveness both in vitro and in vivo. The aim of this review is to evaluate the literature in order to underline the advantages and disadvantages of polyphenols, a class of dietary compounds, as chemopreventive and chemotherapeutic agents. This review also aims to present polyphenols from different perspectives, starting with mechanisms of action and ending with their toxicity. The bigger picture illustrates that polyphenols have great potential in cervical cancer prevention, with strong effects on gene modulation.

## 1. Introduction

**Figure 1:**
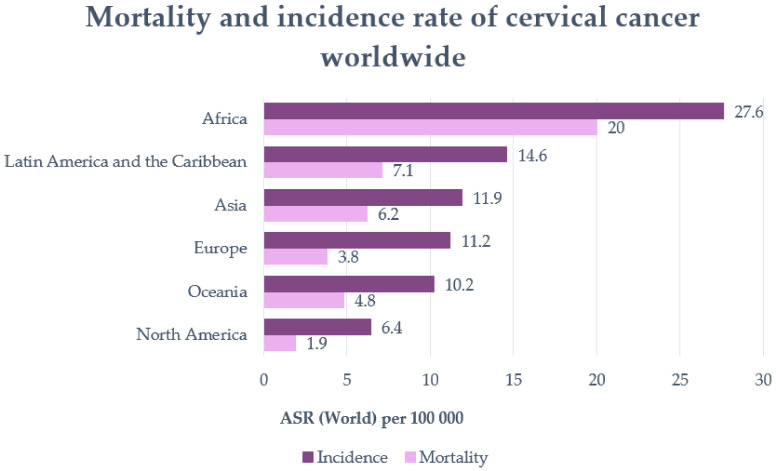
Mortality and incidence rate of cervical cancer worldwide (per 100,000 individuals) (adapted after Khazaei et al., 2019 [2]).

Cancer is one of the leading causes of death, with cervical cancer being the fourth most common cancer type among women worldwide [1]. The incidence and mortality rate varies with geographical location (Figure 1) [2]. Numerous studies have shown that cervical cancer cannot be triggered only by one factor [3]. Besides HPV, which represents the primary risk factor for the development of cervical cancer, socioeconomic status, venereal diseases, reproductive factors, long-term oral contraceptives, smoking, and obesity have also been highlighted as risk factors for this type of cancer [3,4]. In addition, genetic changes and epigenetic aberrations play an important role in the progression of cervical cancer [4]. The most commonly used therapy for cervical cancer is surgery, more specifically pelvic lymphadenectomy and radical hysterectomy. Of course, radiotherapy and chemotherapy are also used to treat this type of cancer. However, all these therapies have shown signs of major side effects such as bleeding, damage to the organs around the surgery, and the risk of clots. Radiotherapy could yield menopause, discomfort, pain with intercourse, or maybe infertility, while chemotherapy may induce cytotoxicity in the whole body, not only in tumoral cells. Furthermore, cisplatin or other drugs that are usually prescribed for cervical cancer can also lead to major side effects or even drug resistance [5].

In the last decade, studies have shown strong evidence that natural compounds such as polyphenols or other phytochemicals can potentially regulate gene expression by targeting different components of the genetic and epigenetic machinery [4]. Although polyphenols are promising anti-cervical cancer agents, their poor solubility and low oral bioavailability obstruct their potential clinical application [3].

## 2. Cervical Cancer

**Figure 2:**
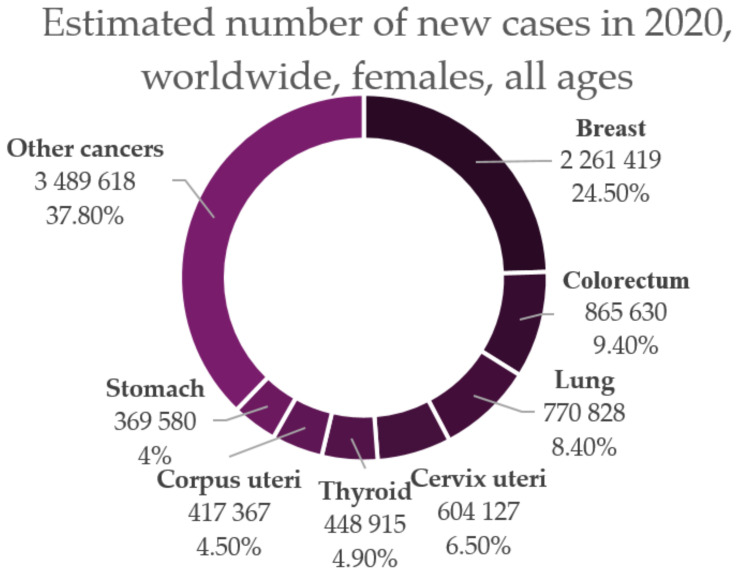
Estimated number of new cases of cancer in 2020 among women of all ages worldwide (adapted after “GLOBOCAN 2020: New Global Cancer Data | UICC.” [1]).

Cervical cancer is a sexually transmitted infection that is caused by high-risk Human papillomavirus (HPV) and according to current data, it is ranked fourth among all cancer types in women worldwide (Figure 2) [6,7]. In the past 30 years, the increasing percentage of young women diagnosed with cervical cancer has ranged from 10% to 40%. Of all the women diagnosed, the age range at which the incidence is the highest is 20–50 years [8].

Regarding the methods of preventing the occurrence of cervical cancer, there are two major methods currently used: anti-HPV vaccination and cervical cancer screening. HPV vaccination prevents over 95% of HPV infections with HPV16 and HPV18 types, while screening detects the early curable phase of cancer, decreasing the mortality associated with cervical cancer. That is the reason why in less developed countries, in which screening and vaccination is not that accessible, about nine out of ten women (89%) die from cervical cancer [8].

In addition to cisgender women, who are the main subjects of cervical cancer studies, it is important to spread awareness about transgender people who may also be victims of cervical cancer because cancer does not discriminate between cis and trans-individuals [9]. In this context, transgender is an umbrella term that describes a diverse group of individuals whose gender identity differs from their sexual identity. Someone who is born a woman but identifies as a man is called a female-to-male, transmasculine, or transgender man. Someone who is born male but identifies as a woman is called a male-to-female, transfeminine, or transgender woman [10]. Both these parts of the LGBTQ+ spectrum can be diagnosed with cervical cancer because not all trans people want to undergo gender-affirming treatment or have the resources to do so. According to many reports, transmen who retain their female genitalia are more likely to miss their screening or other health services (9.2% fewer transmen patients were up to date on their cervical cancer screening than ciswomen patients) because they may not seek out or be included in the target list for screening [11]. This is one of the most important reasons why transmen can be at a higher risk of gynecological cancers including cervical cancer [12]. In contrast, transwomen have a considerable lower risk of cervical cancer than ciswomen because they do not have a proper cervix, as a “neo-cervix” is made of a different type of cells compared to the cervix of ciswomen [8].

## 3. HPV: Structure, Pathogenicity and Transformation Activity

HPV is a member of the Papillomaviridae family and appears to be one of the most common viral pathogens that can lead to sexually transmitted infections worldwide [13,14]. 

**Figure 3:**
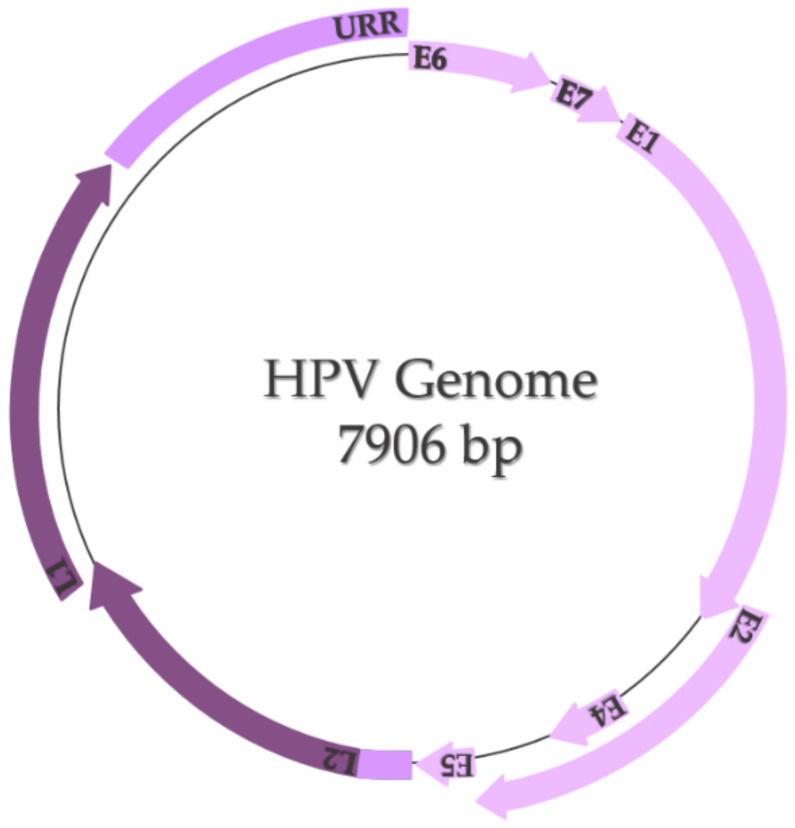
HPV genome (adapted after Bowden and Kyrgiou, 2020 [18]).

The HPV genome is represented by a small double-stranded and highly conserved DNA with a molecular weight of 5 × 10^6^ Daltons and contains approximately 7906 base pairs, including two coding regions (E and L) and one non-coding region called the long control region or upstream regulatory region (URR) (Figure 3) [15,16]. The E region encodes six early proteins (E1, E2, E4, E5, E6, and E7), three of them being regulatory proteins (E1, E2, and E4) and three of them being oncoproteins (E5, E6, and E7) that participate in the processes of replication and transformation of the host cells [17]. E1 and E2 are specifically involved in transcription and replication, E4 is involved in the process of virion release, E5 modulates cell proliferation, and E6 and E7 control the principal signaling pathways in the host cell [18]. The L region encodes two late proteins (L1 and L2), which are the structural proteins that form the viral capsid; L1 is responsible for the major viral capsid and L2 is responsible for the minor viral capsid [15,17,18]. The long control region contains the viral open reading frame (ORF) and the promoter and enhancer elements that modulate the viral DNA replication and transcription [17,18].

In nature, HPVs represent only five out of all 39 genera of the Papillomaviridae family: alpha, beta, gamma, mu, and nu papillomaviruses, the alpha-papillomavirus being the one that causes genital warts [14]. In terms of viruses’ serotype, every HPV is genetically different based on the nucleotide sequence of the gene that encodes the L1 protein; thus, the classification is based on the chronological order of the dates on which they were found. Another form of classification is related to the carcinogenic potential of the HPVs-group 1 carcinogens (carcinogenic for humans), group 2A carcinogens (probably carcinogenic for humans), and group 2B carcinogens (potentially carcinogenic for humans) [16]. Of all high-risk HPVs, the most carcinogenic are HPV16 (approximately 50% of all cervical cancers are associated with this strain) and HPV18 because they are primarily involved in squamous epithelial lesions [19].

Infection with HPV is highly associated with sexual activity, non-sexual transmission, and transmission via fomites. Once the virus enter the body, it manages to interact with squamous epithelial cells via surface receptors such as α-6 integrines or heparin sulfate proteoglycans, infect them, and get access to basal cells during any form of abrasion. Here, in the basal cell, the expression of the E1 and E2 genes is induced, which means that the rolling circle replication begins [20]. The viral genome is integrated in the host genome, leading to the loss of E2’s ORF. This aspect is very important because E2 is the transcriptional repressor of E6 and E7 oncogenes. With that being said, when the replication is over, the E2’s ORF is missing and thus the E6 and E7 genes are overexpressed, which leads to cell transformation [21]. Another possible way to prevent the E2-mediated repression is by methylation of E2 binding sites within the URR [22]. After the transformation, L1 and L2 proteins will make the capsid and the mature virus ready to be released by the E4 protein to other cells [20].

**Figure 4:**
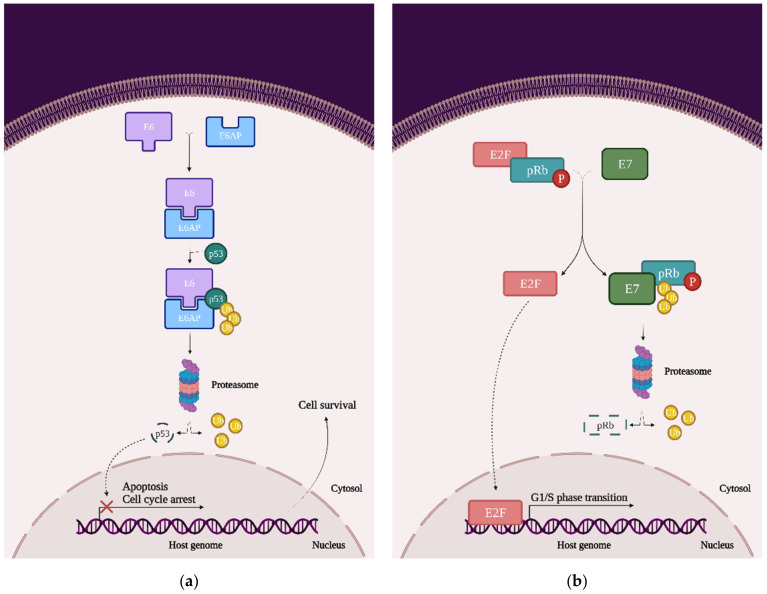

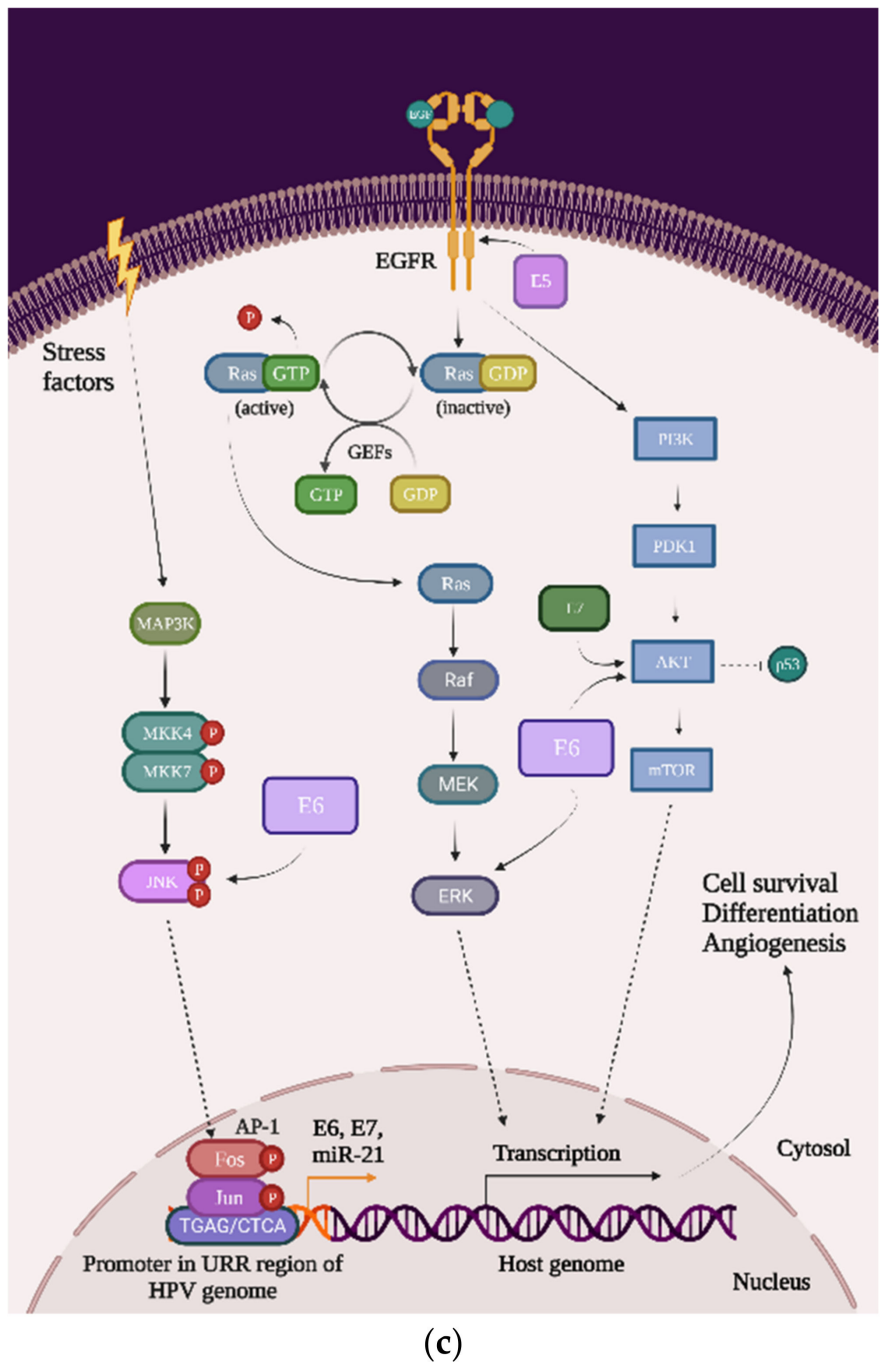
The impact of HPV viral proteins on the main signaling pathways responsible for cell survival, proliferation, and differentiation: (**a**) the effect of the E6 oncoprotein on the p53 tran-scription factor; (**b**) the effect of the E7 oncoprotein on the pRb tumor-suppression protein; and (**c**) the effects of the E5, E6, and E7 oncoproteins on EGFR phosphorylation, the PI3K/Akt/mTOR pathway, JNK, ERK, and the AP-1 complex.

From a molecular perspective, in order for HPV to transform the host cell, it must initiate a series of genetic changes. Thus, in order to prevent or even treat cervical cancer, it is necessary to understand not only the virus’ mechanism of invasion but also the mechanism by which the virus transforms the host cell into a cancer cell. To achieve this goal, it is necessary to visualize the overall image of the cell, focusing on the cellular signaling pathways responsible for the cell cycle, cell growth, and proliferation and induction of apoptosis. More specifically, it is necessary to analyze the possible mutations in the main proto-oncogenes and tumor suppressor genes (TSG), or the possible complexes that may occur due to the presence of HPV in the host cell (Figure 4):

- *p53*: This transcription factor is involved in processes such as the cell cycle arrest, apoptosis, or induction of DNA damage response. In cervical cancer cells, HPVs are capable of inducing p53 ubiquitination via forming a complex between p53, the E6 oncoprotein, and the ubiquitin E3 ligase E6-associated protein (E6AP). This process will lead to p53 degradation by the proteasome and inevitably to chromosomal instability and avoidance of apoptosis and cell cycle arrest (Figure 4a) [23];
- *pRb and pocket proteins*: The retinoblastoma protein (pRb) is a tumor-suppressor protein and, together with p107 and p130, they form “the pocket proteins” that control the cell cycle. pRb needs to bind to the E2F transcription factor in order to reduce its expression and keep the cell in a G1/S phase. In cervical cancer cells, HPV’s E7 protein binds to the pRb-E2F complex and releases the E2F. E2F will be now expressed, which means that the cell will pass the G1/S phase and the pRb will be eventually degraded by the proteasome (this mechanism of degradation requires the binding to the cullin-2 ubiquitin ligase complex) (Figure 4b) [22];
- *EGFR*: The epidermal growth factor receptor (EGFR) is a transmembrane protein that contains an extracellular region that binds the ligands (such as the epidermal growth factor (EGF)), a transmembrane region, and an intracellular region, namely homodimers that have the catalytic site. Once the ligand is bound to the receptor, the EGFR homodimers autophosphorylate and activate some cellular pathways such as the mitogen-activated protein kinase (MAPK), phosphoinositide-3-kinase (PI3K), and protein kinase B (AKT). Primarily, EGFRs are involved in the signaling pathway that controls cell proliferation, differentiation, angiogenesis, and migration and survival, and the high expression of EGFR’s genes is associated with a poor prognosis in many cancer types. In cervical cancer, the HPV oncoprotein E5 increases the phosphorylation level of EGFRs, which lead to hyperproliferation (Figure 4c) [23];
- *PI3K/Akt/mTOR*: This signaling cascade targets some of the most important and complex intracellular processes, which are triggered by a series of internal and external stimuli such as cell proliferation, apoptosis, energy metabolism, growth, and migration. In cervical cancer cells, both E6 and E7 oncoproteins have the ability to upregulate the expression of PI3K and Akt, which will upregulate the expression of mTOR. Once mTOR is overexpressed, it will enhance cell proliferation, which will lead to carcinogenesis (Figure 4c) [24,25];
- *MAPK/JNK*: c-Jun N-terminal kinase (JNK) is a member of the subfamily Ser/Thr kinases (and is one of the three main classes of MAPK) and consists of ten isoforms encoded by three different genes, namely JNK1 (four isoforms ubiquitously expressed), JNK2 (four isoforms ubiquitously expressed), and JNK3 (two isoforms). The JNK signaling pathway can modulate oncogenic and tumor suppressive functions but it depends on the tissue in which it exercises its function. In cervical cancer cells, the E6 oncoprotein manages to increase JNK1/2 phosphorylation via the PDZ-binding motif. With that being said, when JNK1/2 is phosphorylated, c-Jun expression is activated, which induces the proliferation and expression of viral oncoproteins (Figure 4c) [23,26];
- *MAPK/ERK*: The extracellular signal-regulated kinase (ERK) represents another one of the three major classes of MAPK. The ERK pathway is associated with a large variety of processes such as proliferation, senescence, angiogenesis, survival, apoptosis, and differentiation. In cervical cancer cells, the E6 oncoprotein can upregulate the expression of ERK and both the E6 and E7 oncoproteins can regulate hypoxia-inducible factor 1α (HIF-1α), interleukine-8 (IL-8), and the vascular endothelial growth factor (VEGF), which can lead to high rates of proliferation, differentiation, and angiogenesis (Figure 4c) [23,27,28,29];
- *AP-1*: The activating protein-1 (AP-1) is an early transcription factor that plays an essential role in the transcription regulation of the HPV genome. Unlike normal cells, cervical cancer cells have high levels of AP-1 binding activity. AP-1 also represents a transcription factor family, with c-Fos and c-Jun as one of the crucial members. They bind to many consensus DNA-binding sequences (TGAG/CTCA) that are located in the promotor region of the genes and organize a series of gene expression processes of transformation, invasion, and metastasis. Furthermore, in cervical cancer cells, AP-1 upregulated microRNA miR-21 expression, which can contribute to an oncogenic potential. In cervical cancer cells, AP-1 binds to the HPV promoter located in the URR and thereby increases the expression of E6 and E7 oncoproteins, leading to carcinogenesis (Figure 4c) [23,30,31,32].

## 4. Cervical Cancer Treatments

Once the diagnosis is made, it is important to determine the patient’s clinical stage so that the treatment can be chosen appropriately [33]. By cancer staging, the degree of disease progression is determined, this being measured from 0 to IV, wherein 0 is the pre-cancerous/non-invasive stage and IV is the stage in which tumor cells can be found in certain areas of the body (the tumor is metastasized) [34]. 

For patients in the early stage (clinical stage IA), the generally accepted treatment is surgical. If patients show signs of relapse, it is helpful to receive chemotherapy at the same time. For patients in more advanced stages of the disease (clinical stages from IB2 to IVA), it is recommended to receive concomitant chemotherapy and radiotherapy [33]. For the chemotherapy, the most used chemical antitumor agent is cisplatin, which can be used as a unit agent or can be administered in combination with other agents including ifosfamide, paclitaxel, gemcitabine, topotecan, or vinorelbine [35]. 

Besides that, there are other therapies administered on a large scale such as monoclonal antibodies. A good example is Nivolumab, an anti-PD-1 monoclonal antibody which is capable of targeting the programmed death ligand 1 (PD-L1). In this context, in order for HPV to be maintained in the host cell and potentially develop a tumor, it needs to overexpress PD-L1. Therefore, once the immunotherapy starts, PD-L1 will be blocked by Nivolumab and the antitumor activity in cervical cells will be exerted [33].

Although classic treatments offer patients an extension of life without tumor progression, these invasive and non-invasive procedures also have their own disadvantages, namely the side effects. Many existing studies managed to present the dark side of concomitant chemoradiotherapy, highlighting the toxicity of the cervical cancer therapy on a large number of organs. Fundamentally, the main idea of the studies was that chemoradiotherapy can disrupt the long-term quality of life [36]. The following main side effects have been reported:

- *urologic complications*: bladder compliance, incontinence of urine, dysuria, hematuria, hemorrhagic cystitis, ureteral stricture, bowel obstruction, ureteric fibrosis, and vesicovaginal and ureterovaginal fistula [36,37,38];
- *gastrointestinal symptoms*: diarrhea, malaise, ulceration, fecal urgency, tenesmus, fecal incontinence, and rectal bleeding [36,37,39];
- *cardiovascular symptoms*: pulmonary embolus [38];
- *hematological toxicity*: anemia, neutropenia, and thrombocytopenia [38,39];
- *sexual dysfunctions*: sexual discomfort, pain with penetration, hot flashes, vaginal dryness and bleeding, and reproductive concerns [36];
- *lymphedema*: especially lower-extremity lymphedema [36];
- *psychosocial problems*: mood and stress disorders, reduced daily activities and decreased performance of social activity, depression and anxiety, body image concerns, and fear of recurrence [36].

Therefore, due to these side effects caused by the treatment of cervical cancer, the paradigm has changed over time and the emphasis has begun to be placed not on treatment but on prevention. Although this disease is largely preventable, in low-income or middle-income countries, this type of cancer occurs because of a lack of screening and HPV vaccination programs [40]. 

As far as is known, diet has a major impact on specific neoplasia and the most important diet is based on phytochemicals. Polyphenols, for example, can be used in the prevention and treatment of cervical cancer because of their properties, including the induction of apoptosis in HPV cells, inhibition of DNA synthesis, growth arrest, and modulation of signal transduction pathways [41].

In addition to its chemopreventive properties, polyphenols also play the role of sensitizers of cancer cells. Considering the toxic side effects of current therapy, achieving radio and chemo-sensitization of cancer cells, along with minimal toxicity overall, represents a goal in the oncological field. Furthermore, our work will present more information regarding polyphenols and both their chemopreventive and chemotherapeutic properties, studied on cervical cancer cell lines.

## 5. Polyphenols

Polyphenols compose one of the most diverse groups of plant metabolites [42] and, along with vitamins and enzymes, they represent a defense mechanism against oxidative stress caused by excess reactive oxygen species (ROS) [43,44,45]. These compounds are also the subject of many studies that focus on oxidative stress and its associated diseases such as cancer, diabetes, asthma, cardiovascular diseases, or even aging. All these studies aimed to find new chemopreventive agents that are less toxic than classical therapies but still effective [43,46]. 

In order to reveal their characteristics, most specifically their anticarcinogenic properties, scientists tested the polyphenols on multiple cell lines. The results emphasis that the phenolic compounds have a lot of health-promoting properties including antiproliferative, antineoplastic, proapoptotic, and anti-inflammatory activities [43,46,47]. These are the reasons why natural compounds have gained more attention over the last years, especially in the field of cancer. Polyphenols have great potential to act as anticancer drugs not only because of their properties but also because of their availability and toxicity statuses [48].

### 5.1. Polyphenols’ Classification

**Figure 5:**
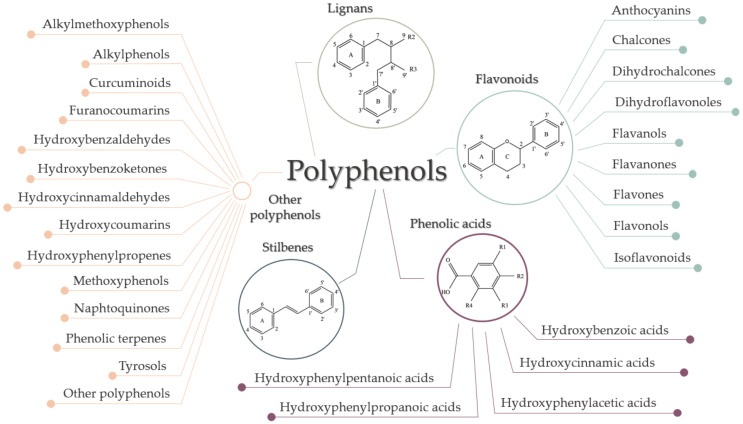
Classification of polyphenols.

Although they are characterized as compounds with phenolic structural features, this group of dietary phenolics is diverse and contains sub-groups of phenolic compounds. Therefore, polyphenols are classified by their chemical structure (the number of phenol rings or the structural elements that bind these rings to one another) in four major classes: flavonoids, phenolic acids, lignans, and stilbenes [43,44,49]. Each class sums up a series of subclasses, all mentioned in Figure 5. In addition to these four classes, there are many more compounds that cannot be categorized into a specific class [4,44,49].

### 5.2. Polyphenols’ Mechanisms of Action

Fundamentally, the primary role of polyphenols is to protect plants from photosynthetic stress, ROS, and consumption by herbivores. In addition, polyphenols represent a significant part of the human diet, namely flavonoids, and phenolic acids are the most common in our food. Numerous studies have been performed to understand the molecular mechanisms underlying their chemotherapeutic and chemopreventive properties on cervical cancer lines [50]. Following these studies, three major mechanisms of action were determined: modulation of gene expression by involving epigenetic pathways, suppression of cancer stem cells (CSCs), and modulation of the cellular redox status [51,52].

#### 5.2.1. Modulation of Gene Expression by Involving Epigenetic Pathways

Epigenetic refers to a series of reversible heritable changes that are not encoded in the DNA but have an important role when it comes to modulating the gene expression [50]. The three main epigenetic mechanisms studied in mammalian cells are DNA methylation, post-transcriptional gene regulation by non-coding RNA (microRNAs/miRNAs), and histone modification [53]. 

In the normal cells, all induce chromatin remodeling, which leads to variations of cell phenotypes, but, when these mechanisms get to be aberrant, they can induce alterations in the expression of oncogenes and tumor suppressor genes. These alterations can accumulate throughout life and eventually affect the transcript stability, the complete nuclear organization of the genetic material, and lastly can initiate tumorigenesis [50,53]. 

Studies have shown that polyphenols are involved in epigenetic processes that influence the behavior of tumor cells and, not only that, they also are involved in the protection of normal cells by enhancing the cytotoxicity of other therapies in tumor cells [4,54].

##### DNA Methylation

DNA methylation is believed to be the most studied epigenetic modification in mammalian cells [55]. It occurs more specifically to regulate tumor growth and the development of carcinogenesis by activation of oncogenes, in addition to silencing TSGs [4]. Consequently, this mechanism of epigenetic machinery is responsible for X-chromosome inactivation and genomic imprinting of even the repression of repeated elements [53]. DNA methylation appears in CpG islands, which are areas of DNA in which a cytosine nucleotide is followed by a guanine nucleotide in 5′ → 3′ direction. These CpG islands are located mostly in promoter regions of the genes as well as in intergenic regions or in regions of large repetitive sequences [4,50]. The key enzymes that modulate this process are DNA methyltransferase enzymes (DNMT). They transfer a methyl group to the 5′ carbon position of cytosine to form 5-methylcytosine [56].

**Figure 6:**
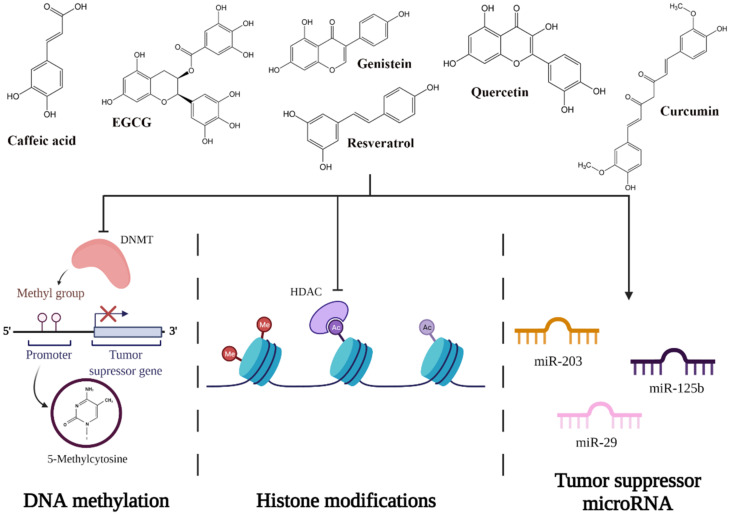
Potential mechanisms of action of polyphenols on epigenetic pathways.

Natural polyphenols, such as resveratrol, genistein, quercetin, or epigallocatechin-3-gallate (EGCG), induce changes in the levels of DNMTs by the direct or indirect effect on DNMT activity (Figure 6) [4,57]. EGCG, for example, is well-known for its capacity to bind directly to the DNMTs, inactivating the enzymes [57]. Conversely, quercetin not only acts as a competitive inhibitor for various members of DNMT families and downregulates their gene expression, but it can restore the expression of TSGs by reducing the methylation of their promoters [57].

##### Histone Modifications

The process of histone modification occurs because of the translational and post-translational modifications (PTMs) [56]. These PTMs occur mostly within the histones’ N-terminal tail or within their globular domain and include a variety of processes such as acetylation, biotinylation, phosphorylation, ubiquitination, SUMOylation, ADP ribosylation, proline isomerization, citrullination, butyrylation, propionylation, and glycosylation [57]. Thus, these mechanisms interrupt the chromatin organization and add new binding sites in a specific region of chromatin. The key enzymes that modulate these processes are histone acetyltransferases (HATs), histone deacetylases (HDACs), and histone methyltransferases (HMTs) [56].

Dietary polyphenols can modulate histone modification to prevent cancer by inhibiting HDAC [4,51]. Quercetin (inhibits HDAC2, HDAC4, HDAC7, and HDAC8), genistein (inhibits HDAC6 and tyrosine kinases), caffeic acid, and curcumin are the most well known for their capacity of inhibiting HDACs (Figure 6) [58].

##### Non-Coding RNA: MicroRNA

MicroRNAs (miRNAs) are short, non-coding single-stranded RNA fragments that regulate cellular processes through transcriptional repression and degradation of messenger RNA (mRNA) [59]. miRNA binds to the mRNA by sequence-specific base pairing with both 3’-untranslated regions of the target fragment and regions that can be realized by complete or partial complementary [5,57,59]. When miRNA binds to mRNA via imperfect complementary, translational repression will occur not only in a single fragment of mRNA, but also in tens to hundreds of different mRNAs. When miRNA binds to mRNA via perfect complementary, degradation of mRNA will occur [59]. In order to achieve this, miRNA needs a protein complex called the RNA-induced silencing complex (RISC), which is a complex that degrades the mRNA after the miRNA is fixed [57]. In vitro and in vivo studies emphasized that miRNAs are classified both as tumor suppressors and oncogenes, and their expression is downregulated or upregulated based on the tumor needs [60].

Various studies have shown that polyphenols also have a significant role in modulating mRNA function. One of the promising polyphenols in cervical cancer therapy is EGCG, a phytochemical found in green tea that has the potential to induce apoptosis in cervical cell lines. Zhu et al. tested the effect of EGCG on multiple cervical cell lines and noticed that in the CaSki cell line, EGCG upregulates miR-203, miR-125b, and mir-29, which are tumor suppressors in cervical cancer cells (Figure 6) [61,62,63,64,65].

#### 5.2.2. Modulation of the Cellular Redox Status

Oxidative stress represents an imbalance between two processes, namely the formation and elimination of oxidative species such as superoxide anion, hydroxyl radical, and hydrogen peroxide [66]. These compounds are primarily the result of cytochrome P450 and peroxisome actions, and when they accumulate, they lead to a series of dysfunctions in the cell [67]. In normal cells, antioxidant compounds come in handy because they are capable of restoring the redox homeostasis by modulating the formation and degradation of ROS [68]. In contrast, in cancer cells, oxidative stress plays an important role in the epigenetic reprogramming of the expression of oncogenes and TSGs [51]. It is well known that cancer cells are usually under greater oxidative stress than normal cells but this property may be an advantage for the discovery of pro-oxidants that induce selective cytotoxicity in tumor cells [68].

What is fascinating about polyphenols is the fact that they can manifest their pro-oxidant properties only in tumor cells, not in normal ones, by decreasing cell viability precisely through the promotion of ROS [68,69]. A good example is curcumin; it has the potential to increase ROS levels in cervical cancer cells, which triggers endoplasmic reticulum stress (ER stress). Once ER stress is initiated, it induces ER stress-mediated apoptosis through activation of the C/EBP Homologous Protein CHOP (transcription factor involved in apoptosis) [70].

#### 5.2.3. Suppression of Cancer Stem Cells

Cancer stem cells (CSCs) are a small subpopulation of tumor cells that play an important role in many processes such as tumorigenicity, tumorigenesis, defining tumor size, the speed of development, trans-differentiating into vascular endothelial cells or other stromal cells associated with the tumor, self-renewal, slow-cycling capacity, metastasis, and the level of regression following treatment [71,72,73]. Furthermore, in contrast with the differentiated cancer cells, CSCs are known for their low ROS levels, more efficient DNA repair responses, and promotion of glycolysis and autophagy [51]. Many studies state that the elimination of CSCs can represent a permanent cure for cancer [73]. The problem is that in cervical cancer, CSCs are associated with chemoradio-resisting properties [52,72].

Shin et al. [52] managed to prove that polyphenols might be a natural alternative to chemoradiotherapy, contributing to tumor cell destruction. More specifically, they showed that pterostilbenes are not only a promising therapy for cervical CSCs, but they also are greater inhibitors than other polyphenols such as resveratrol [52]. Their study demonstrated that pterostilbenes can:

- induce cycle cell arrest at the S/G1 phase via the induction of p53 and p21 (both TSGs), and the reduction of cyclin E1 and cyclin B1;
- induce apoptosis via the downregulation of Bcl-2 and Bcl-XL (antiapoptotic proteins), and ROS-mediated activation of caspase-3 and caspase-9;
- inhibit MMP-2 and MM-9 expression (matrix metalloproteinases) [52].

Despite all this information, polyphenols are involved in many signaling pathways, which will not be detailed in this review. However, in Table 1, the most used polyphenols in cervical cancer research and their main effects observed in both in vitro and in vivo studies are summarized.

### 5.3. A Perspective on Polyphenols’ Toxicity

So far, we have deduced that polyphenols are indeed promising agents against cervical cancer but their accelerated metabolism and reduced bioavailability are obstacles in accomplishing their activity. That is the reason why, in order to achieve the desired results, a high dose of polyphenols is needed [157]. Nevertheless, there is still a question that should be addressed: is a high dose of polyphenols equal to a high rate of absorption or a high efficiency?

It is well known that in any drug development process, a crucial part is represented by the toxicological study [158]. As for polyphenols, it seems that dose, which is linked to bioavailability in most of the cases, can be just as beneficial as it can be harmful for the body [159]. Most of the studies confirm that a higher dose of polyphenols is usually linked with toxicity, but why is this association widely recognized? The shortest and simplest answer is the notion of hormesis [160]. From a biological perspective, hormesis is an adaptive response to stress. When the cell is exposed to a lower concentration of stress-inducing agents, some signaling pathways will be activated in order to confer resistance to higher concentrations of the same agents or for other ones. From a chemical perspective, hormesis is a phenomenon that is characterized by a biphasic dose-response curve. Essentially, a chemical (in our case, a polyphenol) can act as a stimulant when given in small doses and as a toxic agent when given in high doses [161].

In this context, some of the risky doses of polyphenols that have been used in cervical cancer research, together with their harmful effects, are summarized in Table 2.

## 6. Conclusions and Perspectives

To sum up everything that has been stated so far, it is safe to say that polyphenols can be cataloged as a double-edged therapeutic agent with both advantages and disadvantages.

The main advantage of polyphenols is their ability to fight with cancer cells in many ways. Polyphenols have the capacity to modulate the expression of many oncogenes/TSGs and therefore to change the cell dynamics in order to finally maintain the integrity of the host cell intact. Another advantage is that polyphenols can be found in a large variety of plants in both higher or lower concentrations, which gives them their quality of dietary compounds used in cancer prevention.

The main disadvantage is their low bioavailability, which makes them hard to work with because they need high concentrations in order to do their job. This leads us to the second disadvantage, which concerns the polyphenols’ toxicity: in high concentrations, polyphenols can induce toxicity in the organism. With a moderate plant-based diet, though, this disadvantage can be avoided. These disadvantages limit and/or compromise the effectiveness of the compound. Recent studies emphasized that approximately 50–60% of cancer patients from the US choose to use plant-based or alternative medicine rather than chemoradiotherapy; thus, there is a pressing need to find solutions in order to enhance the efficacy of treatment and reduce the side effects [74].

A considerable solution is to encapsulate the polyphenols in various systems such as nanoparticles. Accordingly, the polyphenols can be protected from the destructive action of external media and be carried in an improved delivery system that can optimize and maximize their performance by modifying their composition, morphology, and size by reducing the side effects and overcoming drug resistance [171]. Therefore, the use of nano-sized phytochemicals is desired because they have high biocompatibility, biodegradability, and stability in the biological environment, and also enhance drug specificity, improve absorption rates, and reduce drug degradation and systemic toxicity [74].

Although this might be a promising approach, manufacturing this kind of nanotechnology remains an issue for clinical success. In the future, the emphasis will be on the safety of nanocarriers, on achieving effectiveness by improving the pharmaceutical properties of therapeutic molecules, and on the determination of optimal physicochemical parameters [171].

## Figures and Tables

**Table 1:** Summary of the most used polyphenols in cervical cancer therapy. ↑ indicates upregulation/induction/increasing, while ↓ indicates downregulation/reduction/decreasing.

Compound	Major Dietary Sources	Study Type	Cell Line/Model Organism	Dose	Mechanism/Efficacy	References
**FLAVONOIDS**						
***Flavonols***						
Fisetin	Cucumber, onion, persimmon, strawberry, and apple	In vitro	HeLa	0–80 µM	- ↓ Proliferation - ↑ Apoptosis - ↑ Caspase-8-/caspase-3-dependent pathway - ↓ Tumor growth	[41,74]
		In vivo	Immunodeficient nude mice	2–4 mg/kg		
Galangin	*Alpinia officinarum*	In vitro	HeLa	25–100 µM	- ↓ Proliferation - ↓ Cell migration - ↓ Glyoxalase-1 - ↓ Nfr-2 - ↑ ROS - ↑ Cell death	[75,76]
Isorhamnetin	*Ginkgo biloba*, *Persicaria thunbergii*, *Oenanthe javanica*, and *Hippophae rhamnoides*	In vitro	HeLa	10–80 μg/mL 1–1000 μM	- Destruction of microtubule function by ↓ tubulin expression - ↓ Proliferation - ↓ Telomerase activity - ↓Cdc25C - ↓Cdc2 - ↓Cyclin B1 - ↓Bcl-2 - ↑ AMT-Chk2 pathway - ↑ Cell cycle arrest at G2/M - ↑ Bax - ↑ Apoptosis - ↑ p-Cdc25C - ↑ p-Cdc2	[77,78,79,80]
Kaempferol	Onions, oranges, and parsley	In vitro	HeLa SiHa CaSki C33A HaCaT	2.5–100 μM	- ↓ Proliferation - ↓ Cyclin B1 - ↓ CDK1 - ↓ NF-κB nuclear translocation - ↓ Bcl-2 - ↑ Bax - ↑ Cell cycle arrest at G2/M	[41,81,82]
Morin	White mulberry, osage orange, apple guava, old fustic, strawberry, almond hull, figs, sweet chestnut, onions, jack fruit, and red wine	In vitro	HeLa	4–500 μM	- ↓ Binding of HPV E6 oncoprotein to FADD and caspase-8 - ↓ Proliferation - ↓ CDK1 - ↓ Cdc25C - ↓ Survivin - ↓ Cyclin B1 - ↓ CHK2, Bcl-2, Bcl-xL, AMPK, cIAP-1, cIAP-2, PKCε, and NF-kβ mRNA expression - ↑ Morphological changes - ↑ Cell cycle arrest at G2/M - ↑ p53 - ↑ p21 - ↑ Wee 1 - ↑ Apoptosis - ↑ Death receptors pathway related genes mRNA expression - ↑ Bax, Bad, cytochrome c, Apaf-1, and caspases-9 genes mRNA expression - ↑ PI3K, AKT, mTOR, P70S6K, and Smac genes mRNA expression - ↑ ROS	[83,84,85]
Myricetin	Cranberry, dock, sweet potato leaves, chard, swiss, broad beans (immature seeds), rutabagas, garlic, blueberry, peppers (hot chili, green) blackberry, lotus root, and lemon	In vitro	HeLa	10–100 μM	- LDH release - ↓ Mitochondrial membrane potentials - ↑ Caspase-3 - ↑ Apoptosis - ↑ Cell cycle arrest at G0/G1	[86,87,88,89]
Rutin	Asparagus, buckwheat, apricots, apples, cherries, grapes, grapefruit, plums, oranges, and tea	In vitro	HeLa	2.5–100 µg/ml	- ↓ Proliferation - ↓ Tumor growth	[82,90,91,92]
		In vivo	K14-HPV16 mice		- ↓ COX-2 - ↓ Leukocytic infiltration in HPV-induced lesions	
Quercetin	Onion, kale, leek, broccoli, buckwheat, red grapes, tea, and apples	In vitro	HeLa	25–110.38 ± 0.66 µM	- Restores TSG expression fold change - ↓ DNMTs - ↓ HDAC - ↓ HMT H3K9 - ↓ 5’CpG promoter methylation of TSGs - ↑ Apoptosis - ↑ ROS - ↑ Cell cycle arrest at G2/M	[41,69,82,93]
***Flavones***						
Apigenin	Onions, oranges, tea, some seasonings, Chinese cabbage, bell pepper, garlic, bilimbi fruit, guava, wolfberry leaves, and local celery	In vitro	HeLa	0.1–10 µM	- ↓ Bcl-2 - ↑ Cell cycle arrest - ↑ Apoptosis - ↑ p53	[41,94,95]
Baicalein	Dry root of *Scutellaria baicalensis*	In vitro	HeLa SiHa C33A	2050 µM	- ↓ Colony-forming capacity - ↓ Invasion - ↓ p21/p27 - ↓ Hedgehog/Gli signaling pathway - ↓ NF-κB pathway - ↓ TGFβ pathway - ↓ Proliferation - ↓ mTOR/p70S6K signal pathway - ↓ AKT/mTOR signal pathway - ↓ Notch-1/Hes-1 (Hes-5) - ↓ Cell migration - ↓ miR-19a-3p - ↑ CyclinD1 - ↑ Cell cycle arrest at G0/G1 - ↑ Apoptosis - ↑ Bax/Bcl-2 ratio - ↑ Fas - ↑ FasL - ↑ Caspase-8 - ↑ E-cadherin	[96,97,98,99,100,101,102,103]
		In vivo	Nude mice (with cervical cancer xenograft)	10 mg/kg/day	- ↓ Long non-coding RNA	
			Kun Ming mice with mouse U14 cervical cancer cell line	20–40 mg/kg	- ↓ Tumor weight - ↑ Thymus weight and spleen weight - ↑ Bax/Bcl-2 ratio	
Chrysin	*Scutellaria discolor,* propolis, honey, mushroom, and honeycomb	In vitro	HeLa	10–160 μg/mL 10–30 μM	- ↓ Akt signaling - ↓ Proliferation - ↑ Apoptosis - ↑ Caspases - ↑ p38 - ↑ NFkB/p65 - ↑ Cell cycle arrest	[104,105,106]
Eupatorin	*Orthosiphon stamineus*, *Lantana montevidensis*, and *Tanacetum vulgare*	In vitro	HeLa CaSki	0–320 µM	- ↓ Cleaved caspase-3 expression - ↓ Proliferation - ↓ Hedgehog signal pathway - ↓ Angiogenesis - ↑ Cell cycle arrest at G2/M - ↑ Cyclin B1 - ↑ Cyclin D1 - ↑ Ki67 - ↑ p53 - ↑ p21 - ↑ Bax - ↑ Caspase-mediated apoptosis	[107,108,109,110,111]
Luteolin	Bird’s eye chili, onion leaves, and bilimbi fruit	In vitro	HeLa	20–80 µmol/L	- ↑ Apoptosis by both extrinsic and intrinsic apoptotic pathways	[41,95]
***Isoflavonoids***						
Calycosin	*Radix astragali*	In vitro	HeLa CaSki SiHa C33A	10–50 µM	- ↓ Invasion - ↓ miR-375 - ↓ Cell viability - ↑ Apoptosis	[112]
Daidzein	Soy	In vitro	HeLa	6.25–100 mmol/L	- ↑ Human telomerase catalytic subunit mRNA decreased - ↑ Cell growth arrest - ↑ Cell cycle arrest - ↑ Telomerase activity	[41,113,114]
Genistein	Soy, beans, chickpeas, alfalfa, and peanuts	In vitro	HeLa CaSki	100 µM	- ↓ Cell migration by modulating MMP-9 and TIMP-1 - ↑ Cell growth arrest - ↑ Apoptosis - ↑ Cell cycle arrest at G2/M - ↑ Activity of cisplatin (a chemotherapeutic agent)	[4,41,61,69,115]
		In vivo	Agouti mice Sprague- Dawley rats References			
Isoliquiritigenin	*Glycyrrhiza inflata* and *Glycyrrhiza radix*	In vitro	HeLa CaSki SiHa C33A	10–80 µM	- ↓ Bcl-2 - ↓ HPV E6 oncoproteins - ↓ p-p53 - ↓ cdc25C - ↓ cdc2 - ↓ Cyclin A - ↓ Cyclin B - ↑ Apoptosis - ↑ Cell cycle arrest at G2/M - ↑ Caspase-3 - ↑ Caspase-8 - ↑ Cleavage of caspase-9 - ↑ Caspase-12 - ↑ PARP - ↑ Bax - ↑ p53 - ↑ p21 - ↑ Cytochrome c - ↑ p-eIF2α - ↑ GRP78 level - ↑ p-Chk2	[116,117]
Puerarin	*Pueraria lobata*, *Pueraria thomsonii*, and *Pueraria tuberosa*	In vitro	HeLa	0.5–2 mM	- ↓ Cell proliferation - ↓ P13K, ↓ p-Akt, and ↓ p-mTOR - ↑ Apoptosis	[118]
***Flavanones***						
Hesperetin	Citrus fruit	In vitro	SiHa	25–400 µM	- ↓ Cell viability - ↓ Bcl-2 - ↑ Extrinsic and intrinsic apoptosis - ↑ Cytochrome c - ↑ Cleaved caspase-3 - ↑ Cleaved caspase-8 - ↑ Cleaved caspase-9 - ↑ p53 - ↑ Bax - ↑ FADD - ↑ Fas - ↑ Cell cycle arrest at G2/M	[41,69,82,119]
Hesperidin (Hesperetin-7-O-rutinoside)	Citrus fruit	In vitro	HeLa SiHa CaSki C33A	25–400 µM	- ↓ HPV E6 oncoproteins - ↑p53 - ↑ Cell cycle arrest at G2/M - ↑ Cell growth arrest	[4,69,120,121]
		In vivo	Xenograft mice, rats	650 µM	- ↓ DNMT - ↓ HDAC - ↓ Cell proliferation - ↑ Apoptosis - ↑ ER stress - ↑ Glucose uptake - ↑ ASK1/JNK pathway - ↑ ROS	
Naringin	Citrus fruit	In vitro	SiHa HeLa	250–2000 µM	- ↓ Cell proliferation - ↓ Caspase-1 - ↑ Apoptosis through both death-receptor and mitochondrial pathways - ↑ Cell cycle arrest at G2/M - ↑ Caspase-3 - ↑ Caspase-9 - ↑ p53 - ↑ Bax - ↑ Fas - ↑ FADD	[122,123]
***Anthocyanins***						
Cyanidin	Berries, red fruits, some cereals, and root vegetables	In vitro	HeLa	1.89 µg/mL	- ↓ Proliferation - ↑ ROS - ↑ Peroxides	[41,124,125,126]
Peonidin	Berries, red fruits, some cereals, and root vegetables	In vitro	HeLa	0.84 µg/mL	- ↓ Proliferation - ↑ ROS - ↑ Peroxides	[125]
***Flavanols***						
EGCG	Green tea	In vivo	Humans	200 mg/os	- Controls and promotes IL-23-dependent DNA repair - Modulates growth factor-mediated pathway, the mitogen-activated protein kinase-dependent pathway, and ubiquitin/proteasome degradation pathways - ↓ Carcinogenic signal transduction pathways - ↑ Cytotoxic T-cell activities	[43]
***Chalcones***						
Butein	*Toxicodendron vernicifluum*, *Semecarpus anacardium*, *Dalbergia odorifera*, *Caragana jubata*, and *Rhusverniciflua* sp.	In vitro	HeLa MCF-7 ME-180 SiHa C33A	5–100 µM	- ↓ Colony-forming capacity - ↓ Cell viability - ↓ Cell migration - ↓ XIAP - ↓ cIAP-1 - ↑ Cell growth arrest - ↑ Apoptosis - ↑ DNA damages - ↑ Cell cycle arrest at G2/M - ↑ Caspase-3 - ↑ Caspase-8 - ↑ Caspase-9 - ↑ ROS - ↓ Tumor growth	[127,128,129]
		In vivo	HeLa xenograft mouse	5 mg/kg		
Xanthohumol	*Humulus lupulus*	In vitro	CaSki HeLa	10–40 µM	- ↓ Bcl-2 - ↓ XIAP - ↓ Cell proliferation - ↓ Mitochondrial membrane potential - ↑ Cell cycle arrest at S - ↑ Caspase-3 - ↑ Caspase-8 - ↑ Caspase-9 - ↑ PARP - ↑ p53 - ↑ AIF - ↑ Apoptosis by both extrinsic and intrinsic apoptotic pathways - ↑ DNA fragmentations - ↑ Morphological changes - ↑ TRAIL-R2 protein levels	[130,131,132]
***Dihydrochalcones***						
Phloretin	Fruit, leaves, and roots of apple tree	In vitro	HeLa CaSki SiHa	20–60 µM	- ↓ Cell viability - ↓ Cell migration - ↓ Invasion - ↓ Colony-forming capacity - ↓ Cathepsin S - ↓ MMP-2 - ↓ MMP-3 - ↓ Self-renewal ability - ↓ ALDH1 activity - ↓ Protease activities of cervical cancer-initiating cells - ↓ Lung colonization - ↓ Tumor growth - ↓ Angiogenesis	[133,134]
		In vivo	SiHa xenograft mouse	Up to 100 µM		
***PHENOLIC ACIDS***						
***Hydroxybenzoic acids***						
Ellagic acid	Longan (*Dimocarpus longan*), litchi (*Litchi chinensis*), walnuts, pecans, cranberries, raspberries, strawberries, grapes, and peaches	In vitro	HeLa SiHa C33A	10–30 µM	- ↓ Cell viability - ↓ HPV E6 oncoprotein - ↓ STAT3 signaling - ↓ Cyclin D1 - ↓ Bcl-x1 - ↓ Mcl-1 - ↓ Cell migration - ↓ Invasion - ↑ Apoptosis - ↑ Cell cycle arrest at G1/S/G2 - ↑ p53 - ↑ Bax - ↑ Caspase3 - ↑ Caspase9 - ↑ Cell growth arrest	[135,136,137,138,139]
		In vivo	Mice	50–100 mg/kg/day		
Gallic acid	Blackberry, raspberry, walnuts, chocolate, wine, green tea, and vinegar	In vitro	HeLa HTB-35 HUVEC	10–40 µg/mL	- ↓ Cell viability - ↓ Proliferation - ↓ Invasion - ↓ Angiogenesis - ↓ Cytotoxicity on normal cells (HUVEC) - ↑ ROS and GSH depletion	[41,69]
Punicalagin	*Punica granatum*	In vitro	HeLa ME-180	12.5-200 µM	- Modulating MMP-2, MMP-9, TIMP-2, and TIMP-3 - ↓ Cell viability - ↓ Bcl-2 - ↓ Cell migration - ↓ β-catenin signaling pathway - ↓ Mitochondrial membrane potential - ↑ ROS - ↑ Apoptosis - ↑ Bax - ↑ Caspase-3 - ↑ Caspase-9 - ↑ p53	[140,141]
***Hydroxycinnamic acids***						
Caffeic Acid	Coffee, fruits, vegetables, and olive oil	In vitro	HeLa ME-180	50 μg/mL	- ↓ Mitochondrial membrane potential - ↑ Lipid peroxidative markers (thio-barbituric acid reactive substances, conjugated dienes, and lipid hydroperoxide) - ↑ ROS - ↑ Apoptotic morphological changes	[82,142]
Ferulic Acid	Cereal grains, particularly the outer parts of the grain	In vitro	HeLa ME-180	10 µg/mL	- ↓ Cell viability - ↑ Efficacy of radiotherapy probably through ↑ ROS	[69]
***STILBENES***						
***Stilbenes***						
Pterostilbene	Grapes, blueberries, red wine, peanuts, and some medicinal plants	In vitro	HeLa PC1	30 µM	- ↓ HPV E6 Oncoprotein in vivo and in vitro - ↓ VEGF Protein in vivo - ↑ Tumor development by ↑ cell cycle arrest and ↓ tumor growth - ↑ Cleaved Caspase-3	[5,143,144]
		In vivo	HPV E6 Mice	1 mM		
Resveratrol	Red wine, grapes, and berries	In vitro	SiHa HeLa C-33A	150–250 µM	- ↓ Proliferation - ↓ Metastatic potential by inactivating phosphorylation of STAT3^Tyr705^ - ↓ HPV E6 oncoprotein - ↓ PCNA protein in vivo - ↓ VEGF protein in vivo - ↑ Apoptosis - ↑ Fission proteins Fis1 and Deo1 - ↑ ER stress - ↑ MiR-326/pyruvate kinase M2	[4,41,69,144,145,146]
		In vivo	Xenograft Mice			
***OTHER POLYPHENOLS***						
***Curcuminoids***						
Curcumin	Rhizome of the medicinal plant turmeric (*Curcuma longa*)	In vitro	SiHa	15 μM in SiHa	- ↓ HPV18 transcription by selectively ↓ AP-1, which reverses the expression dynamics of c-fos and fra-1 - ↓ Telomerase activity - ↓ Ras - ↓ ERK signaling pathways - ↓ Cyclin D1 - ↓ COX-2 - ↓ iNOS activity - ↓ Mitochondrial pathway - ↓ HPV E6 oncoprotein - ↑ p21 - ↑ ROS	[139,147]
			CaSki			
			HeLa	25 μM in HeLa		
***Hydroxybenzoketones***						
Paeonol	*Cynanchum paniculatum*, and *Paeonia suffruticosa*	In vitro	HeLa	0.1–0.6 mg/mL	- ↓ Cell migration - ↓ Invasion - ↓ 5-lipoxygenase - ↓ Proliferation - ↓ PI3K/AKT signaling pathway - ↑ Apoptosis - ↑ Morphological changes - ↑ ROS - ↑ Cytochrome c - ↑ Bax/Bcl-2 ratio - ↑ Caspase-3	[148,149]
***Hydroxycoumarins***						
Scopoletin	*Scopolia* sp.	In vitro	HeLa SiHa C33A DoTc2	0–100 μM	- ↓ PI3K/AKT signaling pathway - ↓ Cell migration - ↑ Cell growth arrest - ↑ Caspase-3 - ↑ Caspase-8 - ↑ Caspase-9 - ↑ Apoptosis - ↑ DNA damages - ↑ Cell cycle arrest at G2/M	[150]
***Naphtoquinones***						
Juglone	*Juglans mandshurica*	In vitro	HeLa CaSki C33A	10–100 μM	- ↓ Cell viability - ↓ Proliferation - ↓ Cell migration - ↓ Invasion - ↑ Cell cycle arrest at G2/M - ↑ Morphological changes - ↑ Apoptosis - ↑ Cytochrome c - ↑ Caspase-3 - ↑ PARP - ↑ p-JNK - ↑ p-c-Jun	[151,152,153]
***Phenolic terpenes***						
Carnosic acid	Chinese medicinal herbs	In vitro	CaSki SiHa	10–30 μM	- ↓ Proliferation - ↑ Cell growth arrest - ↑ Apoptosis - ↑ Caspase-3 - ↑ Caspase-9 - ↑ Cell cycle arrest at G2/M - ↑ ROS	[154]
		In vivo	CaSki xenograft mouse	20–30 mg/kg	- ↓ Tumor growth	
***Other polyphenols***						
Salvianolic Acid B	*Salvia miltiorrhiza*	In vitro	HeLa	20–200 μM	- ↓ Cell viability - ↓ TNF levels - ↑ Apoptosis	[155,156]

**Table 2:** Polyphenols’ main toxic effects based on their high administration doses.

Compound	Dose	Model Organism	Toxicological Effects	References
**FLAVONOIDS**				
***Flavonols***				
Quercetin	- 2–4% above the normal dose	Mice	- Chronic nephropathy - ↑ Redox cycling of catechol-estrogens and estradiol-induced tumorigenesis	[162,163]
	- ≥100 µg/mL		- ↑ DNA damages	
***Isoflavones***				
Genistein	- ≥500 ppm	Mice Humans	- Modification of anti-luteinizing hormone in premenopausal women - Manifestation of sexual maturation in infants - ↓ Thyroid peroxidase activity - ↓ Fertility	[159,162,164]
***Anthocyanins***				
Proanthocyanidin	- ≥10 g/kg	Mice	- ↓ Growth and digestibility	[162,165]
	- ≥100–500 µg/ml	Chick cardiomyocytes	- ↑ LDH - ↑ ROS - Cell death by ↑ caspase-3	
***Flavanols***				
EGCG	- ≥200 µM - ≥200–400 mg/kg ip	Mice	- Toxicity in liver, kidney, and intestine via ↑ ROS - ↑ DNA damage	[163,166,167]
***PHENOLIC ACIDS***				
***Hydroxycinnamic acids***				
Caffeic acid	- ≥20 g/kg - ≥5–10 g/kg	Mice	- Carcinogenic - Tumor promoter	[159]
Ferulic acid	- ≥500 mg/kg	Mice	- Carcinogenic to liver	[159]
***STILBENES***				
***Stilbenes***				
Pterostilbene	- ≥250 mg/day	Humans	- ↓ Bicarbonate, which can cause minor acid effects in blood	[168]
Resveratrol	- ≥25 µM (in vitro) - ≥1 g/day (in vivo)	Humans	- Nephrotoxicity - ↓ White blood cells - ↓ IL-6 - ↓ TNF - ↓ Cytochrome P450 isoenzymes - ↑ Alanine aminotransferase - ↑ DNA damage and proteolysis - ↑ CVD biomarkers - ↑ Angiogenesis - ↑ EMT transition - ↑ Invasion - ↑ Metastasis	[169]
***OTHER POLYPHENOLS***				
***Curcuminoids***				
Curcumin	- ≥0.5 mg/day	Humans	- Nausea, diarrhea, headache, rash, and yellow stool - ↑ Serum alkaline phosphatase - ↑ LDH	[170]

## Abbreviations

HPV	Human papillomavirus
URR	upstream regulatory region
Rb	retinoblastoma gene
ORF	open reading frame
E6AP	E6-associated protein
TSG	tumor suppressor genes
pRb	retinoblastoma protein
EGFR	epidermal growth factor receptor
EGF	epidermal growth factor
ROS	reactive oxygen species
MAPK	mitogen-activated protein kinase
PI3K	phosphoinositide-3-kinase
AKT	protein kinase B
JNK	c-Jun N-terminal kinase
ERK	extracellular signal-regulated kinase
PD-L1	programmed death ligand 1
CHOP	C/EBP Homologous Protein
CSCs	cancer stem cells
DNMT	DNA methyltransferase
EGCG	epigallocatechin-3-gallate
HAT	histone acetyltransferase
HDAC	histone deacetylase
HMT	histone methyltransferase
PTMs	post-translational modifications
miRNA	microRNA
mRNA	messenger RNA
RISC	RNA-induced silencing complex
ER stress	endoplasmic reticulum stress
CSCs	cancer stem cells
